# Supplementary material for: Postoperative Cerebral Venous Sinus Thrombosis Following a Retrosigmoid Craniotomy—A Clinical and Radiological Analysis
Source: Brain Sci. 2023 Jul 7;13(7):1039. doi: 10.3390/brainsci13071039 (PMC10377583; doi:10.3390/brainsci13071039)
Supplement: Supplementary file 1 [file brainsci-13-01039-s001.zip › brainsci-2473197-supplementary.pdf]

Supplementary Table S1. Surgical and postoperative characteristics of 130 patients included in the study. Values are n (%) or the median (range), as indicated. N/A – not applicable

Characteristic	Data	p–value
**Sex**		0.02
male	51 (39.2%)	
female	79 (60.8%)	
**Age (years)**		N/A
median (range)	46.0 (20 – 82)	
1st, 3rd quartile	36, 57.75	
**Diagnosis**		N/A
Vestibular schwannoma, including:	109 (83.85%)	
neurofibromatosis type 2	3 (2.31%)	
vestibular schwannoma and meningioma	2 (1.54%)	
Meningioma, including:	15 (11.54%)	
Petroclival	7 (5.38%)	
Sphenopetroclival	2 (1.54%)	
Glossopharyngeal schwannoma	1 (0.77%)	
Facial schwannoma	1 (0.77%)	
Glioblastoma	1 (0.77%)	
Cavernous malformation	1 (0.77%)	

Metastasis	1 (0.77%)	
Unknown histopathology	1 (0.77%)	
**Side**		0.86
right	64 (49.3%)	
left	66 (50.7%)	
**Tumor**		N/A
maximal size: median (range), 1st, 3rd quartile (mm)	31.5 (9 – 57), 22.25, 39	
volume: median (range), 1st, 3rd quartile (cm^3)	11.25 (0.18 – 59.9), 3.82, 21.57	

Supplementary Table S2. Characteristics of patients diagnosed with clinical cerebral venous sinus thrombosis. PMH —past medical history, SSS —superior sagittal sinus, VS —vestibular schwannoma

**Characteristics**	**Patient 1**	**Patient 2**	**Patient 3**	**Patient 4**
Age, sex	41, female	46, female	66, male	42, female
Diagnosis	VS	meningioma	metastasis	VS and meningioma
Side	right	right	left	left
Sinus dominance	right	left	right	left
Tumor size, mm (volume, cm^3)	18 x 11 x 12 (1.24)	14 x 12 x 10 (0.88)	40 x 35 x 33 (24.2)	25 x 15 x 20 (3.9)
Distance to the transverse sinus	-10.05	-2.4	-6.5	-10.55
Exposure of the transverse sinus	exposed	exposed	exposed	exposed
Distance to the sigmoid sinus	-7	0	-5.55	-2.7
Exposure of the sigmoid sinus	exposed	hidden	exposed	exposed
Transverse sinus thrombosis	no	yes	yes	yes
Sigmoid sinus thrombosis	no	yes	yes	yes

Junction of sinuses thrombosis	no	yes	yes	yes
Distant thrombosis of other sinuses	yes, SSS	no	no	no
Intraparenchymal hematoma	no	yes	yes	no
Injuries of the sinuses	no	no	no	no
Risk factors	no	no	smoking, oncologic PMH	overweight/obesity, oncologic PMH
Postoperative headaches refractory to regular medical treatment	yes	yes	no	no
Outcome	death	discharged home	discharged to neurology department	discharged home

Supplementary Table S3. Literature review on the cerebral venous sinus thrombosis after cerebellopontine angle surgery. Abbreviations: CPA – cerebellopontine angle, MF – middle fossa, N/A – not applicable, RS – retrosigmoid, TL – translabyrinthine, TP – transpetrosal, VS – vestibular schwannoma

Author, year	Study type	Incidence (%)	Diagnosis	Approach
Benjamin, 2019 [14]	prospective, single center	24/74 (32.4)	tumor near sinus	RS, TL, suboccipital, supratentorial
Guazzo, 2019 [18]	prospective, multicenter	21/54 (38.9)	VS	TL
Ohata, 1998 [20]	retrospective, single center	7/143 (4.9)	undisclosed	presigmoid TP
Keiper, 1999 [33]	retrospective, single center	4/105 (4.7)	undisclosed	RS, TL
Moore, 2014 [13]	retrospective, single center	5/43 (11.6)	CPA tumor	RS, TL, MF
Apra, 2017 [16]	retrospective, single center	12/180 (6.7)	posterior fossa tumor	midline, paramedian, lateral suboccipital
Jean, 2017 [34]	retrospective, single center	10/52 (19.2)	undisclosed	TL, posterior petrosectomy
Abou-Al-Shaar, 2018 [7]	retrospective, single center	7/116 (6.03)	VS	RS, TL, MF
Shew, 2018 [24]	retrospective, single center	22/127 (17.3)	CPA tumor	TL, RS, MF

Yen Kow, 2020 [26]	retrospective, single center	20/126 (15.9)	CPA tumor	RS, TL, subtemporal
Orlev, 2020 [25]	retrospective, single center	26/538 (4.8)	posterior fossa tumor	RS, TL, other
Ziegler, 2020 [35]	retrospective, single center	1/128 (0.8)	VS	RS, TL
Gerges, 2021 [15]	retrospective, single center	10/61 (61.4)	VS	TL
Krystkiewicz, 2022 [36]	retrospective, single center	26/116 (22.4%)	VS	RS
current study	retrospective, single center	61/130 (46.9%)	posterior fossa tumor	RS

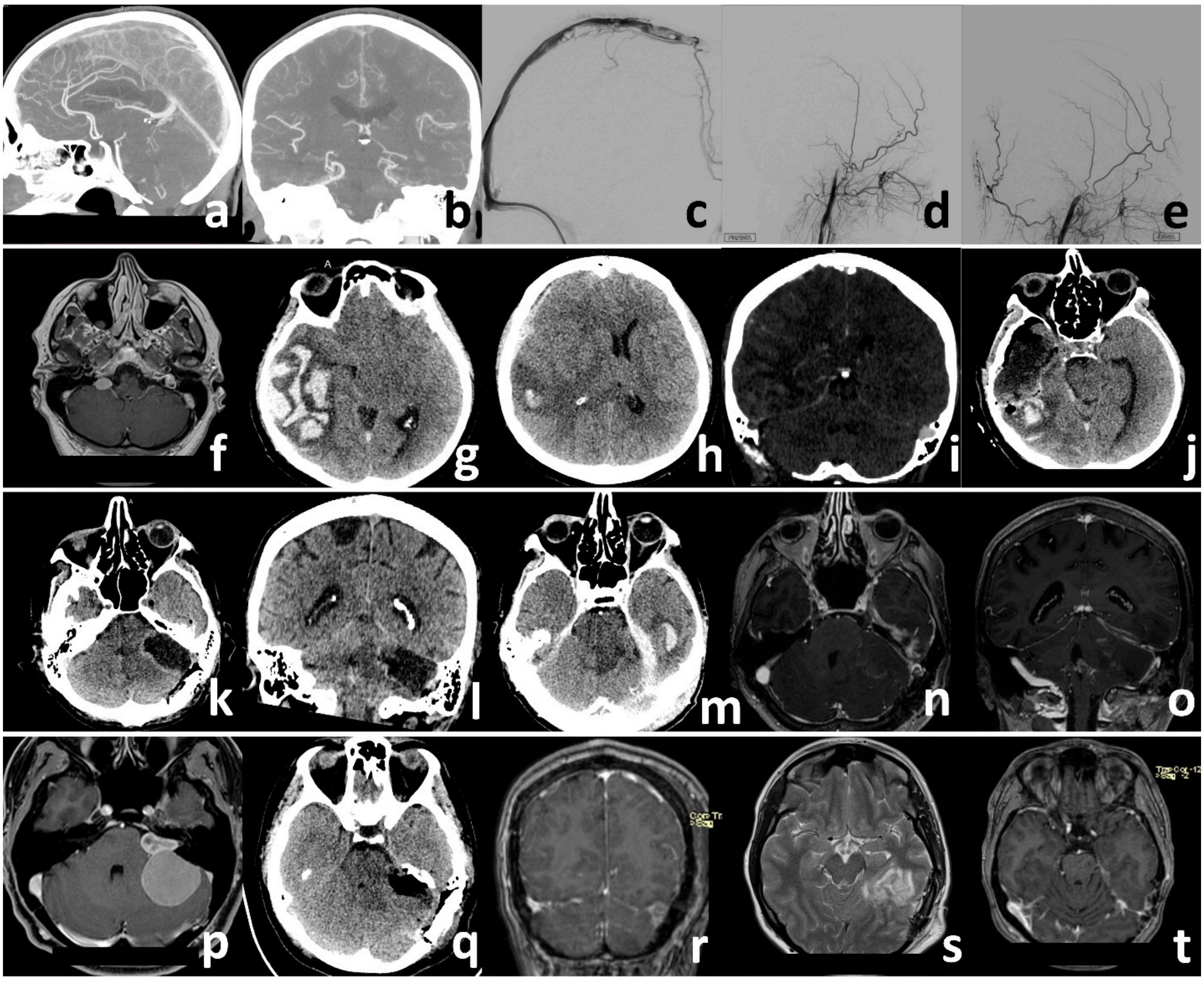

Supplementary Figure S1. Radiologic images of patients diagnosed with clinical cerebral venous sinus thrombosis.

Patient 1 (a – e) Sagittal and coronal maximum intensity projections of CT angiography (a and b) of first patient visualize superior sagittal sinus thrombosis after retrosigmoid craniotomy for a vestibular schwannoma resection. After unsuccessful endovascular treatment (c) the patient died in the intensive care unit. The lateral views of right and left common carotid artery angiography injections (d and e) prior to death are significant for lack of internal carotid arteries flow.

Patient 2 (f – j) Preoperative T1–weighted axial MRI scan (f) visualize a right cerebellopontine angle tumor which was confirmed as a meningioma in the second patient. An intraparenchymal hematoma in the right temporal lobe (g) causing a mass effect and a midline shift (h) is shown in postoperative axial CT images. A coronal CT angiography is significant for right lateral sinus thrombosis. The patient underwent successful surgical removal of a hematoma, as presented on the axial CT scan (j).

Patient 3 (k – o) A satisfactory early postoperative CT axial and coronal scans (k and l) of a patient who underwent left retrosigmoid craniotomy. However, routine postoperative contrast enhanced CT scan visualized a small intraparenchymal hemorrhage in the basal part of the ipsilateral temporal lobe (m). A

subsequent contrast enhanced T1–weighted MRI axial and coronal scans (n and o) confirmed cerebral venous sinus thrombosis.

Patient 4 (p – t) Preoperative contrast enhanced T1 –weighted MRI axial scan (p) shows an unusual coincidence of a meningioma and vestibular schwannoma in the left cerebellopontine angle. Because of neurological deterioration in the second postoperative day the patient had an MRI and was diagnosed with cerebral venous sinus thrombosis, as can be seen on a coronal contrast enhanced T1–weighted MRI scan (r). A small intraparenchymal hemorrhage in the adjacent temporal lobe was visualized on axial T2–weighted MR image (s), which resolved in follow–up (contrast enhanced T1 -weighted MRI scan, t).
